# Systematic Review: Preclinical and Clinical Evidence on MSC Efficacy and Mechanism of Action in Neuropathic Pain Reduction

**DOI:** 10.3390/ijms27052397

**Published:** 2026-03-05

**Authors:** Zahrina Haziqah Harun, Min Hwei Ng, Nor Hazla Mohamed Haflah, Htwe Ohnmar, Yogeswaran Lokanathan, Zhe Kang Law, Amaramalar Selvi Naicker, Soon Yong Thow, Shalimar Abdullah

**Affiliations:** 1 Department of Orthopaedic & Traumatology, Faculty of Medicine, University Kebangsaan Malaysia, Jalan Yaacob Latif Kuala Lumpur, Bandar Tun Razak, Cheras, Kuala Lumpur 56000, Malaysia; zahrinaharun@gmail.com (Z.H.H.); asnaicker@yahoo.com (A.S.N.); 2 Department of Tissue Engineering & Regenerative Medicine (DTERM), Faculty of Medicine, University Kebangsaan Malaysia, Jalan Yaacob Latif Kuala Lumpur, Bandar Tun Razak, Cheras, Kuala Lumpur 56000, Malaysia; angela@hctm.ukm.edu.my (M.H.N.); lyoges@hctm.ukm.edu.my (Y.L.); 3 Faculty of Medicine, Universiti Sultan Zainal Abidin (UniSZA), Kuala Terengganu 20400, Malaysia; htwe.om@gmail.com; 4 Medical Department, Faculty of Medicine, University Kebangsaan Malaysia, Jalan Yaacob Latif Kuala Lumpur, Bandar Tun Razak, Cheras, Kuala Lumpur 56000, Malaysia; 5 Hospital Sultan Ismail, Jalan Mutiara Emas Utama, Taman Mount Austin, Johor Bahru 81100, Malaysia

**Keywords:** mesenchymal stem cells, neuropathic pain, inflammation, immunomodulation, cell therapy

## Abstract

Nerve injury often results in neuropathic pain, marked by spontaneous pain, hyperalgesia, and allodynia. Current treatments have moderate efficacy and have side effects, prompting interest in alternative approaches. Mesenchymal stem cell (MSC) therapy has shown promise in preclinical studies for reducing neuropathic pain and inflammation. However, the precise mechanisms underlying MSC-mediated pain reduction remain unclear. Investigating these mechanisms is crucial for optimizing MSC-based therapies for neuropathic pain. This article provides a brief overview of the MSC administration, animal models of neuropathic pain, and treatment regimens used in 25 preclinical studies, focusing on the potential mechanisms of action underlying the neuropathic pain-reducing effect of MSCs. Importantly, 23 out of the 25 studies demonstrated a reduction in neuropathic pain following MSC therapy, despite differences in MSC sources and treatment regimens. Neuropathic pain relief was associated with decreased inflammation, suggesting that MSCs may act through immune modulation. However, the resolution of inflammation does not always correlate with complete neuropathic pain relief, indicating the involvement of additional mechanisms.

## 1. Introduction

Nerve injury is a common clinical problem that often leads to neuropathic pain, a chronic pain condition characterized by spontaneous pain, hyperalgesia, and allodynia. Neuropathic pain arises from diverse causes, including traumatic nerve injuries, metabolic disorders, viral infections, and autoimmune diseases, affecting the central nervous system (CNS). Its prevalence is estimated at 3–17% of the general population, and it severely impairs patients’ quality of life, contributing to physical disability, psychological distress, and socioeconomic burden [1]. Increasing evidence highlights the role of neuroinflammation as a key driver of neuropathic pain, promoting maladaptive plasticity and abnormal neuronal excitability. Following nerve injury, persistent immune activation and the imbalance of mediators further drive these pathological changes. Pro-inflammatory mediators and cytokines such as IL-1β, TNF-α, and IL-6 activate nociceptors, regulate MAPK and NF-κB pathways, and alter ion channel function. These changes collectively increase neuronal excitability and hypersensitivity, leading to neuropathic pain [2,3].

Currently, first-line treatments for neuropathic pain include gabapentinoids, tricyclic antidepressants (TCAs), and selective serotonin-norepinephrine reuptake inhibitors (SNRIs). Second-line options consist of lidocaine, capsaicin, and tramadol, while third-line treatments for peripheral neuropathic pain include strong opioids (e.g., morphine and oxycodone) and botulinum toxin-A (BTX-A). However, these drugs are moderately effective and are associated with common side effects, such as lethargy, nausea, and constipation. Additionally, these drugs can cause more serious side effects. TCAs may lead to arrhythmias, while SNRIs like duloxetine and venlafaxine are linked to increased blood pressure and cardiac conduction abnormalities [1].

Mesenchymal stem cells (MSCs) are multipotent stromal cells capable of self-renewing and differentiating into diverse types of cells, including bone, neuronal cells, and cardiomyocytes. They are abundantly present in the body and can be isolated from various tissues, like bone marrow, umbilical cords, and adipose tissue [4,5,6]. In addition, MSCs have also been shown to possess immunomodulatory and anti-inflammatory properties [7]. Early studies on peripheral nerve injuries reported that injected MSCs migrate to the dorsal root ganglia (DRG) of injured nerves [8]. Subsequent research demonstrated that MSCs could reduce mechanical allodynia and decrease cold-induced allodynic responses in rats following sciatic nerve injury [9]. These findings have sparked growing interest in their potential use as a therapy for neuropathic pain, a condition strongly linked to excessive nerve inflammation. Unlike current pharmacological treatments, which mainly alleviate pain by modulating nerve signaling or neurotransmitter levels, MSCs can address the underlying causes of neuropathic pain by promoting tissue repair and regeneration. Additionally, their low immunogenicity suggests a lower risk of the common side effects associated with pharmacological treatments. To date, no comprehensive review of the literature has been performed to ascertain the efficacy of MSCs in the management of neuropathic pain.

In this review article, we compile and compare the mounting evidence in preclinical and clinical studies using MSCs to treat or ameliorate neuropathic pain and the possible mechanism of action. This article provides a brief overview of the MSC administration, animal models of neuropathic pain, and treatment regimens used in preclinical studies, with a focus on the exact mechanisms underlying the neuropathic pain-reducing effect of MSCs. Investigating the mechanisms by which MSCs reduce neuropathic pain can provide insights into the underlying biological processes and help optimize the development of MSC-based therapies for neuropathic pain.

## 2. Methods

Relevant research articles were identified from PubMed and Scopus, with no language restrictions. The literature search included the following terms in the title or abstract: (“mesenchymal stem cell” OR “mesenchymal stromal cell” OR MSC) AND (“neuropathic pain”) AND (mechanism OR pathway) over the period 2009–2023. Filters were used to exclude review articles, conference abstracts, and case reports. Research that utilized MSCs as a secondary or supplementary treatment, as well as studies that used MSCs as a vector only, were excluded. Original studies on the therapeutic effect of MSCs on neuropathic pain, regardless of the source of MSCs, route of administration, and animal model, were included. The usage of MSC secretomes or extracellular vesicles (EVs) as a therapeutic intervention was also included. The titles and abstracts of the retrieved references were independently evaluated by two authors (Harun, Z.H. and Ng, M.H.). Full texts were acquired for any references considered potentially relevant by either author. In instances of disagreement, a third reviewer (Abdullah, S.) was consulted to achieve a consensus.

**Figure 1:**
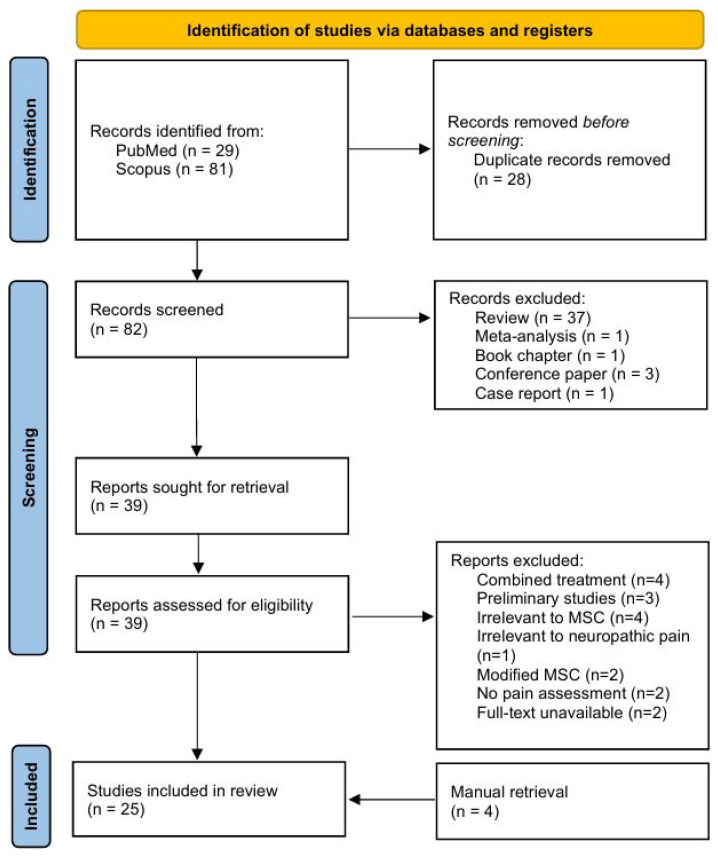
PRISMA flow diagram of identification of studies for systematic review. PROSPERO registration ID CRD420251177390.

For data extraction, three authors (Harun, Z.H., Ng, M.H., and Mohamed-Haflah, N.H.) independently analyzed pertinent data for each study. They examined various aspects of the studies, including animal species, disease model, source of mesenchymal stem cells, route of administration, treatment regimes, and outcomes. The methodological quality of the studies included was evaluated using the Cochrane Risk of Bias (RoB) tool, which assesses five areas: randomization in allocating animals to control or treatment groups, ethical considerations, reporting of baseline characteristics for animal behavioural assessments, blinding of outcome assessor, and declaration of conflicts of interest. Each area was classified as having a “low”, “unclear”, or “high” risk of bias. Any disagreements were resolved through discussion, and if a consensus could not be achieved, a third reviewer (Abdullah, S.) made the final determination. The process of identifying, evaluating, and ultimately including or excluding studies is outlined in Figure 1.

## 3. Results

A total of 110 articles were initially identified through the electronic search. After removing duplicates, 82 articles remained for screening. Of these, 43 articles-including four that were manually retrieved—were selected for full-text evaluation. Following assessment, 25 articles that fulfilled the inclusion criteria were ultimately included in the systematic review (Figure 1).

### 3.1. Data Extraction

The included studies are summarized in Table 1.

### 3.2. Risk of Bias

**Figure 2:**
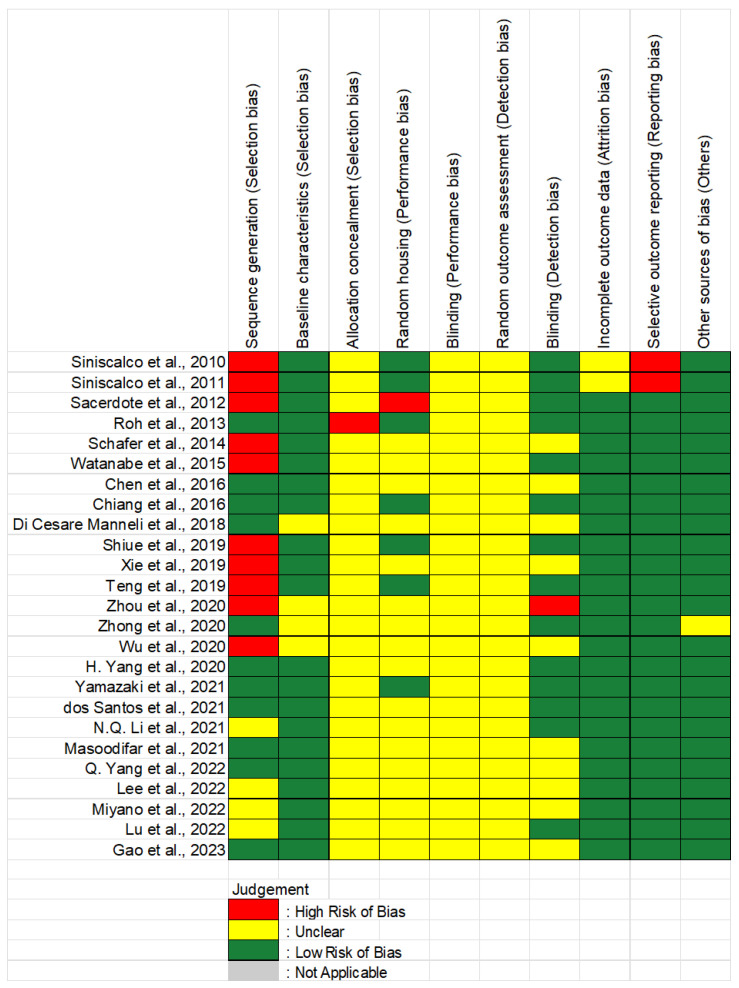
Assessment of risk of bias of the 25 articles [1,2,3,4,5,6,7,8,9,10,11,12,13,14,15,16,17,18,19,20,21,22,23,24,25] included in the systematic review.

The risk of bias (RoB) for each study, assessed using SYRCLE’s RoB tool for animal studies, is shown in Figure 2. Out of the 25 articles included, only 11 studies (44%) implemented a randomization method during the allocation of animals to either the treatment or control group. A significant majority of the studies (84%) reported comparable baseline characteristics, based on behavioral assessments, between the treatment and control groups at the start of the study, while the remaining studies did not provide information on this aspect. In terms of blinding during behavioral assessments, only 14 studies (56%) reported the blinding of the outcome assessor, while 10 studies (40%) did not provide any information on this matter. Additionally, one study [20] explicitly stated that one of the assessors was not blinded during behavioral assessments. In all studies, the methodology did not clearly state whether animal caregivers were also blinded during group allocation, aside from the outcome assessor. This is important because caregivers aware of treatment assignments might handle animals differently, for example by providing extra care to those undergoing certain procedures, which could in turn induce behavioral changes and influence study outcomes. Regarding selective outcome reporting, two studies from the same research group [10,11] did not present all the data they had measured, and no explanation was provided for these omissions, raising concerns about reporting bias. Lastly, for other sources of bias, we reported on animal ethics, with only one study [21] failing to mention ethical considerations regarding the use of animals in research.

### 3.3. Sources of MSC, Route of Administration, and Treatment Regime

Based on the included studies, mesenchymal stem cells (MSCs) were primarily sourced from bone marrow and umbilical cords, mainly from humans, rats, or mice for the former and exclusively from humans for the latter. Other sources included the adipose tissue, amniotic fluid, tonsil, and placenta. They were administered via intrathecal and intravenous injections, with intrathecal delivering MSCs into the cerebrospinal fluid and intravenous injection directly into the bloodstream. Some studies have also administered MSCs directly to the spinal cord around the area of injury. Less common routes included intramedullary, intraperitoneal, intracavity, and dorsal root entry zones. The total number of MSCs per dose ranged from 50,000 to 5,000,000 cells, with 1,000,000 cells being the most used dosage. The concentration of injected MSCs varied from 2500 to 500,000 cells per microliter, with 5000 and 10,000 cells per microliter being common. MSCs were typically administered once within 1 to 21 days after surgery, but some studies used multiple injections (usually two to four). Refer to Table 2 for further details on MSC administration.

Four studies opted to use secreted molecules from MSCs for treatment instead of using the whole cell. One study [25] utilized the conditioned medium, which contained all the molecules secreted by MSCs, including growth factors, cytokines, and extracellular vesicles (EVs). On the other hand, Lu et al. [28] used EVs isolated from conditioned medium, whereas Shiue et al. [17] and Gao et al. [29] used exosomes, a subtype of EVs. Details on the administration of MSC-derived products are summarized in Table 3.

## 4. Discussion

### 4.1. Correlation Between Neuropathic Pain and Inflammation

Among all the studies, only 14 studies provided data on the changes of inflammatory markers after the induction of neuropathic pain.

Out of the 14 studies, 12 found that after surgery, at least one pro-inflammatory marker, such as IL-1β, IL-6, IL-17, IL-33, TNF-α, and COX-2, was upregulated [11,12,14,15,17,20,21,22,26,28,29,30]. The majority of studies [12,14,15,17,22,26,28,29] demonstrated the presence of upregulated pro-inflammatory marker(s) during the first one to two weeks following surgery, which coincided with the peak of neuropathic pain as measured by the paw withdrawal threshold (PWT) or paw withdrawal latency (PWL). This suggests that there may be a correlation between inflammation and neuropathic pain, but the nature of their correlation remains uncertain.

Inflammation is the body’s defence mechanism in response to harmful or foreign stimuli, such as pathogens or tissue injury, and its purpose is to induce healing. When the body is injured, it releases chemical signals that attract white blood cells to the site of injury. These cells then release various chemical factors, such as inflammatory cytokines, to initiate the healing process [35]. Pro-inflammatory cytokines like IL-1β, IL-6, and TNF-α promote inflammation, while anti-inflammatory cytokines like IL-10 and IL-4 inhibit it. Inflammatory enzymes like COX-2 and iNOS are also released to mediate inflammation. The chemical factors involved in mediating inflammation are often used as inflammatory markers by researchers. Thus, the upregulation of pro-inflammatory marker(s) observed in the animals after one to two weeks of surgery is most probably an indication of the ongoing healing process of the damaged tissue. However, it is unclear whether inflammation contributes to or exacerbates neuropathic pain. To determine this, pro-inflammatory marker levels would need to be recorded at different time points during the experiment, ideally during the same time as behavioral assessments. This would enable researchers to determine if there is a direct relationship between inflammation and neuropathic pain. Only one study [22] suggests that inflammation may be directly involved in the development of neuropathic pain, as the authors found that the upregulation of IL-1β, IL-6, and TNF-α followed the same pattern as the development of neuropathic pain in the animals. These pro-inflammatory cytokines gradually increased from day one after surgery and peaked on day seven before gradually decreasing by day 14, but the remained significantly higher than the level observed in the sham group. The same pattern was also observed in the paw withdrawal threshold and paw withdrawal latency readings, suggesting that inflammation may directly contribute to the development of neuropathic pain.

According to several studies [11,20,30], pro-inflammatory markers can remain upregulated for at least a month after surgery. The study conducted by Siniscalco et al. [11] found that IL-1β and IL-17 were still upregulated 30 days after surgery, when neuropathic pain was at its peak. Roh et al. [30] also showed that expression of COX-2, an inflammatory enzyme, was still upregulated after 41 days of surgery, when neuropathic pain was still significant in the animal. These findings indicate that inflammation may play a role in sustaining neuropathic pain. After a traumatic nerve injury, inflammation occurs to signal damage and promote healing. However, the accumulation of pro-inflammatory factors can sensitize nociceptors, including those that are undamaged around the injury site. This is to ensure that the affected tissue or organ is able to rest and heal. As the nerve begins to regenerate, the production of pro-inflammatory factors decreases and anti-inflammatory factors increase, leading to a reduction in inflammation over time. However, in cases of neuropathic pain, inflammation persists, leading to the continuous stimulation and sensitization of surrounding nociceptors, including undamaged ones, resulting in spontaneous activity and contributing to neuropathic pain [36,37,38].

Two studies [13,23] showed that the level of pro-inflammatory cytokines was unchanged after surgery. In the study by dos Santos et al. [23], the levels of IL-1β and TNF-α were only measured 10 weeks after the induction of neuropathic pain, and the findings indicated that the levels of these cytokines were similar between oxaliplatin-induced mice and sham mice. Nonetheless, the oxaliplatin-induced mice continued to display significant mechanical allodynia and thermal hyperalgesia at the 10-week mark. As suggested by the author of [23], inflammation may only play a role in the initial stages of neuropathic pain, rather than during its maintenance or later stages, in this animal model. As no other studies utilizing the oxaliplatin-induced animal model that conducted experiments lasting longer than one month were found, comparison to the results presented by dos Santos et al. [23] cannot be made. However, there are other studies [39,40,41] that have shown that expression of pro-inflammatory cytokines like TNF-α, IL-1β, and IL-6 was significantly upregulated 5 to 21 days after oxaliplatin-induced neuropathic pain, which supports the notion that inflammation is involved during the early stages of neuropathic pain.

Presently, research indicates that inflammation is evident in the initial phases and at the peak of neuropathic pain. Nevertheless, it remains uncertain whether inflammation persists throughout the maintenance phase of neuropathic pain, as current studies and disease models concentrate more on the onset rather than the sustenance of neuropathic pain. Additionally, since these studies assess outcomes at specific time points rather than through continuous monitoring, determining the precise timing and duration of pain relief and inflammation remains challenging.

### 4.2. MSC Efficacy for Neuropathic Pain Reduction: Effect of Dosage, Timing of Administration, and MSC Sources

A total of 23 out of the 25 studies (92% of the studies) showed that the administration of MSCs successfully reduced the incidence of neuropathic pain. MSCs derived from various sources—including the amniotic fluid, bone marrow, adipose tissue, umbilical cord, umbilical cord blood, placenta, and tonsil tissue—have been shown to alleviate neuropathic pain. Effective single doses of MSCs ranged from 50,000 to 5,000,000 cells, while repeated administrations were effective at doses ranging from 500,000 to 2,000,000 cells per injection. For repeated administrations, the lowest effective total number of cells administered was 50,000 cells, and the highest was 8,000,000 cells. Routes of administration that successfully reduced neuropathic pain included intrathecal, intravenous, intramuscular, intraperitoneal, intramedullary, direct delivery to the spinal cord, intracavity injection, and administration to the dorsal root entry zone. In addition, MSC-secreted products have also demonstrated efficacy in reducing neuropathic pain. The lowest effective single injection dose of MSC-secreted products was 1.2 µg total protein, while the highest effective single dose was 12 µg total protein.

#### 4.2.1. Effect of Dosage

In the study by Sacerdote et al. [12], which utilized a chronic constriction injury (CCI) model and adipose-derived mesenchymal stem cells (ASCs) for treatment, the analgesic effect of ASCs was found to be dose-dependent. Administration of 1 × 10^6^ ASCs induced a faster response within 1 day, produced a sustained analgesic effect lasting up to 21 days post-treatment, and demonstrated a stronger analgesic effect, as reflected by paw withdrawal latency (PWL) values in CCI mice reaching levels comparable to those of sham mice by day 7 post-treatment. In contrast, a lower dose of 5 × 10^5^ ASCs provided analgesic effects for only 7 days, and PWL values in treated mice did not reach those observed in sham mice.

In the study by Siniscalco et al. [10], it was observed that a single intrathecal injection of 50 × 10^3^ bone marrow-derived MSCs (BMSCs) in spared nerve injury (SNI) mice decreased mechanical allodynia for at least 11 days. The researchers repeated the experiment a year later using the same animal model (SNI), source of MSCs (bone marrow), and time of injection (4 days post-SNI surgery) but administering 2 × 10^6^ BMSCs intravenously. The results from this study [11] showed a longer-lasting reduction in mechanical allodynia and thermal hyperalgesia, for at least 86 days. However, the duration of the analgesic effect may have been influenced by the experimental design. In the earlier study [10], the effects were assessed for only 16 days after BMSC administration, with the highest analgesic effect observed on the last day. Although it is unclear whether the analgesic effect had reached its peak or would gradually decrease or remain constant over time, both studies [10,11] seem to suggest that a higher dose of MSCs intravenously would result in a longer-lasting analgesic effect compared with a lower dose of MSCs intrathecally. Interestingly, administration of 2 × 10^6^ BMSCs produced a similar onset of analgesic effect as a much lower dose of 50 × 10^3^ BMSCs. In both cases, reductions in mechanical allodynia and thermal hyperalgesia were observed around 6–7 days post-injection [10,11]. However, the delayed response noted in the higher-dose study may be due to its experimental design, which assessed pain behaviours only one week after injection, potentially missing earlier therapeutic effects.

#### 4.2.2. Effect of Timing of Administration

Both Xie et al. [18] and Yang et al. [22] utilized bone marrow-derived MSCs (BMSCs) via intrathecal injection in a CCI model. Xie et al. [18] administered 250 × 10^3^ BMSCs in a single injection, whereas Yang et al. [22] administered a higher dosage of 5 × 10^6^ BMSCs in a single injection. Xie et al. [18] demonstrated successful reduction of mechanical allodynia for at least 21 days, with complete neuropathic pain elimination from the first day to the fifth day following the initial injection (PWT in CCI mice similar to that of the sham mice). In contrast, Yang et al. [22] found that mechanical allodynia and thermal hyperalgesia were reduced for at least 13 days but never completely eliminated (PWT and PWL in CCI rats never reached the same level as the sham rats). These results suggest that a higher dosage of MSCs does not necessarily provide a longer or stronger analgesic effect. The difference in analgesic effect between the two studies could be attributed to the timing of injection. Xie et al. [18] injected BMSCs five days after CCI surgery, when neuropathic pain was at its peak, while Yang et al. [22] administered BMSCs only one day after CCI surgery, when neuropathic pain was still in its early stages and developing. While both timings of administration managed to alleviate neuropathic pain, it is worth considering whether the severity of neuropathic pain might influence the recruitment and/or activation of MSCs. The reason is that as the condition progresses, cells release an increasing number of chemical signals to promote healing, which in turn attracts more MSCs, resulting in a more pronounced or prolonged analgesic effect. Finding the right balance between the dosage and timing of MSC injection is crucial for effective cell therapy. Currently, diagnosing and treating neuropathic pain at its early stages remains a challenge in clinical practice due to the lack of set guidelines and the variability of symptoms among patients. In reality, patients seek treatment only when their pain becomes severe and affects their daily life. Therefore, future studies should focus on optimizing the dosage and timing of MSC injection for later stages of (or chronic) neuropathic pain.

#### 4.2.3. Effect of MSC Sources

Both Wu et al. [32] and Watanabe et al. [31] employed the contusion SCI model and directly injected MSCs into the spinal cord at the site of injury. Wu et al. [32] revealed that the analgesic effect of BMSCs persisted for up to 56 days post-injection, whereas Watanabe et al. [31] showed that the analgesic effect of umbilical cord MSCs (UCMSCs) lasted only 28 days. However, directly comparing the efficacy of MSCs from these two studies is complicated, primarily due to the differences in the tissue sources of the MSCs. MSCs derived from different tissues, such as bone marrow and umbilical cord, have distinct biological properties, which can significantly impact their therapeutic effectiveness [42], particularly in terms of their potential to reduce neuropathic pain. One study [43] demonstrated that UCMSCs possess stronger anti-inflammatory properties than BMSCs, likely due to their higher secretion levels of angiopoietin-1 (Ang-1). Additionally, another study [44] found that the UCMSC secretome may be more effective in mitigating excitotoxicity, while BMSCs are better suited for combating oxidative stress due to the differences in their secretion profiles. These variations could explain the varying analgesic effects reported between UCMSCs and BMSCs in the same animal model, as observed by Watanabe et al. [31] and Wu et al. [32].

A comparison of the studies by Sacerdote et al. [12] and Yang et al. [22] suggests that ASCs may exert a better analgesic effect compared to BMSCs. Administration of 500 × 10^3^ ASCs resulted in a significant reduction of neuropathic pain for up to 7 days [12], whereas the same dose of BMSCs failed to produce any analgesic effect [22]. However, increased doses of BMSCs–1 × 10^6^ and 5 × 10^6^ cells were effective in alleviating neuropathic pain for 11 days, indicating a dose-dependent response [22]. These findings imply that MSCs derived from different tissue sources may possess distinct analgesic effects, potentially due to differences in their secretory profiles. Interestingly, another study by Xie et al. [18] utilizing the same CCI model reported that a single injection of 250 × 10^3^ BMSCs was sufficient to alleviate neuropathic pain for 14 days. This discrepancy highlights the inherent heterogeneity of MSCs, which may not only arise from their tissue of origin but also from factors such as donor variability, cell isolation techniques, and culture conditions—all of which can influence their secretome and therapeutic potential. Currently, there is a limited body of research investigating how MSC heterogeneity influences their therapeutic potential, which presents a significant gap in understanding. Addressing this issue is crucial for optimizing MSC-based therapies and developing standardized protocols that ensure consistent, reliable results across different clinical settings. By exploring the impact of MSC diversity on their functional properties, researchers can identify the most effective MSC subpopulations for specific therapeutic applications, ultimately improving the efficacy of MSC-based treatments for conditions such as neuropathic pain.

#### 4.2.4. Contraindications and Negative/Neutral Findings

Only two studies did not report any pain reduction following MSC treatment. In the study conducted by Schäfer et al. [13], a partial sciatic nerve ligation (PSNL) animal model was utilized, where the sciatic nerve was tightly ligated. However, consistent neuropathic pain in rats was not achieved in their PSNL animal model, as indicated by a significant decrease in PWT compared to the sham group only from the 1st to 7th day and on the 20th day post-surgery, as well as a significant decrease in PWL compared to the sham group only from the 2nd to 4th day and on the 7th day. In contrast, Miyano et al. [27] also used the same animal model and were successful in producing consistent neuropathic pain in rats, as indicated by a significantly lower PWT and weight distribution of paws compared to the sham group from the 4th to 24th day post-surgery. This difference in neuropathic pain establishment between the two studies using the same animal model could be attributed to the method of execution. The tightness of the sciatic nerve ligation, which is subjective to the researcher carrying out the procedure, could vary, resulting in inconsistent outcomes. It is plausible that in Schäfer et al. [13], the ligature was not tight enough to produce consistent neuropathic pain, which could explain the inability to observe changes in PWT and PWL following MSC administration. Additionally, Schäfer et al. [13] admitted that there is a limitation to their study, as their PSNL-induced neuropathic pain was not chronic enough to attract MSCs, which could explain the lack of changes observed following MSC administration.

Another possible explanation is that the treatment regime used may have played a role. Schäfer et al. [13] applied intrathecal injection of 1 × 10^6^ MSCs per injection, whereas Miyano et al. [27] opted for intravenous injection of 5 × 10^6^ MSCs per injection. It is possible that the chosen dosage and administration route were not suitable for the PSNL model, which is a peripheral nerve injury model. Since the MSCs were administered intrathecally, they would need to be reabsorbed from the cerebrospinal fluid (CSF) into the bloodstream before they could reach the target tissue in the periphery, the sciatic nerve in this case. The extent to which MSCs can move from the CSF into the bloodstream is poorly understood due to the ongoing debate among researchers on the production, circulation, and absorption of CSF itself [45,46,47,48]. While one study demonstrated that human bone marrow MSCs transplanted intracerebrally could migrate to the spleen of rats [49], the mechanisms by which MSCs could move from the CSF into the bloodstream have yet to be investigated. Perhaps too few MSCs were able to migrate from the CSF into the bloodstream to reach the sciatic nerve and cause a local response in the study by Schäfer et al. [13], or as hypothesized by the researchers themselves, the PSNL model was not chronic enough to attract MSCs to the injured sciatic nerve.

The study by Roh et al. [30] is another example where administration of MSCs did not result in any reduction in neuropathic pain in rats. Specifically, they found no effect after administering umbilical cord blood-derived MSCs (UCBMSCs) but did observe a reduction in neuropathic pain after administering amniotic epithelial stem cells (AESCs), even though both cell types showed similar survival rates at the injection site. However, it should be noted that this study is the only study that uses a hemisection spinal cord injury (SCI) model and UCBMSCs, making it challenging to directly compare the results with other studies. A different study by Yang et al. [34] also explored the use of UCBMSCs to reduce neuropathic pain, but this time in an animal model of advanced osteoarthritis (OA) induced by monosodium iodoacetate (MIA). Interestingly, they found a significant reduction in mechanical allodynia on the final day of observation, which occurred 21 days after the induction of OA. This suggests that the mechanism of action of UCBMSCs in reducing neuropathic pain might not be as effective in a hemisection SCI animal model. Given that there are only two studies utilizing UCBMSCs, it remains difficult to draw firm conclusions about whether UCBMSCs have varying analgesic effects in different neuropathic animal models, which warrants further research.

#### 4.2.5. Summary

A total of 23 out of the 25 studies reported pain reduction following MSC treatment, with one study noting effects lasting up to day 86. Studies suggest that the efficacy of MSCs in treating neuropathic pain may not only depend on the dosage but also the timing of injection. Achieving a balance between the timing and dosage of injection is crucial to effectively harness the analgesic potential of MSCs. Additionally, the lack of improvement in neuropathic pain observed in two of the selected studies underscores the need for a more reliable marker or potency assay to serve as a release criterion for MSCs, enabling better prediction of their efficacy prior to administration. This further emphasizes the heterogeneity and variability in MSC preparations across different research groups.

### 4.3. Potential Mechanisms of Action of MSCs in Mitigating Neuropathic Pain and Inflammation

#### 4.3.1. Polarization of Microglia from M1 to M2 Type via Various Cell Signaling Pathways

Yang et al. [22] demonstrated that TNF-inducible gene 6 (TSG-6), secreted by BMSC, inhibited the activation of toll-like receptor 2 (TLR2) and myeloid differentiation primary response 88 (MyD88) in microglia. As a result, this reduced the nuclear translocation of p65 of NF-κB in the microglia and subsequently reduced the release of pro-inflammatory cytokines by microglia.

The TLR2/MyD88/NF-κB pathway is a signaling pathway involved in the innate immune response. Following ligand recognition and stimulation, TLR2 recruits adaptor protein MyD88, which then activates the downstream signaling pathway. This ultimately leads to the translocation of p65 NF-κB to the nucleus to bind to target genes for gene expression and consequently the production of pro-inflammatory cytokines, such as TNF-α, IL-1β, and IL-6 [50,51,52]. Inhibition of this pathway by TSG-6 secreted by BMSCs results in reduced inflammation.

Another study by Zhong et al. [21] also showed that BMSCs reduced the phosphorylation of p65 in microglia, leading to a decrease in anti-inflammatory cytokines and an increase in pro-inflammatory cytokines. In this study, rather than TSG-6, the effect was attributed to glial cell-derived neurotrophic factor (GDNF), secreted by BMSCs, which is known for its neuroprotective role in reducing inflammation.

The authors of [21] also demonstrated that BMSCs could reduce inflammation through another pathway known as the PI3K/Akt pathway, in which GDNF promotes the phosphorylation of PI3K and Akt, which leads to the polarization of microglia from the M1 to M2 type. M1 microglia releases pro-inflammatory cytokines, whereas M2 microglia releases anti-inflammatory cytokines. However, it is unknown whether there is a correlation between the activation of the PI3K/Akt pathway and inhibition of NF-κB in reducing inflammation. While existing studies suggest that activation of Akt, upstream or downstream, leads to the activation of NF-κB rather than inhibition [53,54,55], further research is still needed to investigate any potential connection between the activation of the PI3K/Akt pathway and the inhibition of NF-κB, as demonstrated by Zhong et al. [21]. Taken together, these findings suggest that GDNF may exert its regulatory effects through both the TLR2/MyD88/NF-κB pathway and the PI3K/Akt pathway in reducing inflammation.

According to a study by Li et al. [24], BMSCs were also found to reduce the phosphorylation of p65 NF-κB in microglia but through a different pathway. The study showed that inhibiting TRPA1, a member of the TRP cation channels family primarily found in sensory neurons and non-neuronal cells in mammals, using a TRPA1 antagonist resulted in a decrease in the expression of p65 NF-κB. TRPA1 has been reported to play a functional role in pain and neurogenic inflammation, as it is involved in the sensory neural response to chemical irritants such as mustard oil and capsaicin, leading to Ca^2+^ influx and cell depolarization and ultimately the transduction of sensory signals [56,57]. In another in vitro study, it was found that inhibition of TRPA1 via a TRPA1 antagonist reduces the phosphorylation of IκBα, leading to inhibition of the NF-κB pathway [58]. Thus, it is suggested that BMSCs may release an antagonist that acts on TRPA1 to inhibit the phosphorylation of IκBα and subsequently inhibit the activation of NF-κB. This ultimately leads to reduced production of pro-inflammatory cytokines. Further research is needed to confirm this pathway. Additionally, it was also found that the presence of a TRPA1 antagonist resulted in an upregulation of IL-10 production by microglia, indicating that downregulation of TRPA1 may lead to the polarization of M1 to M2 microglia, resulting in an increase in the production of anti-inflammatory cytokines. However, further research is required to confirm this relationship.

Li et al. [24] also demonstrated that treatment with BMSCs can reduce the expression of p38, a member of the mitogen activated protein kinases (MAPK) family involved in inflammatory responses and the production of pro-inflammatory cytokines [59]. While the study [24] did not measure the production of pro-inflammatory cytokines, it did find an upregulation of the anti-inflammatory cytokine IL-10, suggesting that BMSCs may mediate inflammation in neuropathic rats through the p38 MAPK signaling pathway. Numerous pathways have been reported where p38 can regulate NF-κB-mediated gene transcription. In one study [60], the activation of p38 via MEKK1 was found to increase NF-κB-mediated transcription. Therefore, it is hypothesized that BMSCs may reduce NF-κB transcriptional activity by inhibiting p38, leading to an increased production of anti-inflammatory cytokines through M1 to M2 microglia polarization. This pathway would explain the downregulation of both p38 and p65 and the upregulation of IL-10 in LPS-stimulated microglia when co-cultured with BMSCs as shown by Li et al. [24]. Further investigation is required to elucidate this pathway.

The involvement of TRPA1 in the activation of p38 was also investigated in the study [24]. Contradicting results were found, where an in vivo study showed that treatment with a TRPA1 antagonist did not significantly reduce the expression of p38 but in vitro study did. It is speculated that the insignificant reduction in p38 in the in vivo study may be due to the short time frame between TRPA1 antagonist injection and p38 measurement. It is possible that the TRPA1 antagonist had not yet exerted its effect on p38 or the change in p38 level was too low to be detected by western blotting. Therefore, further in vivo study with a longer observation period is needed to establish the correlation between TRPA1 and p38 in this context.

BMSCs may also modulate p38 through other upstream factors. Watanabe et al. [31] conducted an in vivo study that demonstrated reduced expression of p38 and ERK1/2, which were mainly co-localized with microglia, in neuropathic mice following treatment with BMSCs. While p38 is typically associated with cellular stress and apoptosis, the ERK pathway is primarily involved in cell proliferation and differentiation [61]. There is evidence supporting crosstalk between the p38 and ERK1/2 pathways, with one study showing that TGF-β-induced activation of ERK results in upregulation of MKP-1, which subsequently inhibits phosphorylation of p38 MAPK and reduces NF-κB-dependent production of inflammatory mediators [62]. Although these findings seem to contradict those of Watanabe et al. [31], since both p38 and ERK1/2 expression were reduced, it is important to note that many upstream and downstream proteins are involved in each signaling pathway. Thus, BMSCs may regulate the crosstalk between p38 and ERK1/2 via different proteins, either upstream or downstream. Alternatively, there may be no crosstalk between p38 and ERK1/2 underlying the mechanism of action of BMSCs in the study by Watanabe et al. [31]. Further in vivo and in vitro studies are required to elucidate the relationship between these two signaling pathways in the BMSC-mediated relief of neuropathic pain and inflammation.

In a separate in vivo study, Xie et al. [18] observed a decrease in the phosphorylation of ERK1/2 in neuropathic mice following BMSC treatment. Additionally, the level of TGF-β was found to be elevated after BMSC treatment. To further investigate the role of TGF-β in BMSC-mediated neuropathic pain reduction, exogenous TGF-β was injected into neuropathic mice and resulted in reduced pain and decreased phosphorylation of ERK1/2. Conversely, when a TGF-β inhibitor was administered, the observed effects were diminished. These findings suggest that BMSCs may alleviate neuropathic pain by inhibiting ERK1/2 through the upregulation of TGF-β. However, it is not yet clear whether the BMSC itself releases TGF-β or stimulates other cells to release TGF-β, which subsequently inhibits ERK1/2.

In addition to the observed reduction of ERK1/2 expression, Watanabe et al. [31] demonstrated that BMSC treatment resulted in decreased phosphorylation of CREB (cAMP-response element binding protein) in neurons. CREB is a transcription factor known to regulate the expression of various genes in cells, including dopaminergic neurons. Other studies have shown that inhibiting the activation of the ERK/CREB pathway, which can occur via various proteins, can reduce pain in rats [63]. Therefore, it is possible that BMSCs or other cells induced by BMSCs release a protein that can block the phosphorylation of both ERK1/2 and CREB, thereby reducing neuropathic pain.

In the most recent study, Gao et al. [29] found that the TLR2/MyD88/NF-κB signaling pathway in microglia can also be regulated by the RSAD2 gene, which is mostly known for its role in cellular antiviral response. The study demonstrated that exosomes of UCMSCs reduced the expression of RSAD2, TLR2, MyD88, NF-κB, and pro-inflammatory cytokines in the spinal cords of neuropathic rats. In LPS-induced BV2 microglia, knockdown of the RSAD2 gene resulted in reduced expression of TLR2, MyD88, and p65 NF-κB. However, the exact mechanism by which exosomes act on the RSAD2 gene remains to be further investigated.

#### 4.3.2. MicroRNA-Mediated Pathway

The analgesic effect of BMSCs could also be mediated by the microRNAs (miRNAs) they release. MiRNAs are short, non-coding RNA molecules that regulate gene expression by binding to complementary sequences in messenger RNA (mRNA), leading to either degradation of the mRNA or inhibition of its translation. These miRNAs are often encapsulated in extracellular vesicles (EVs) released by MSCs. When recipient cells take up EVs containing miRNAs, these miRNAs can bind to complementary mRNA sequences, thereby influencing gene expression [64,65]. Zhou et al. [20] observed that BMSCs upregulate miRNA-547-5p, which in turn downregulates IL-33 and ST2 in vivo. IL-33 is a cytokine that is primarily studied for its role in immune and inflammatory diseases, whereas ST2 is a receptor for IL-33 [66]. The authors focused on the activity of IL-33 and ST2 in the nervous system and found that IL-33 was downregulated in astrocytes, whereas ST2 was downregulated in neurons of the dorsal root ganglion (DRG) and spinal dorsal horn (SDH). Liu et al. [67] reported that knocking down ST2 in DRG neurons reduces itch response in a mouse model of allergic contact dermatitis (ACD). They also demonstrated that a ST2-neutralizing antibody diminishes robust Ca^2+^ responses in DRG neurons induced by IL-33. The influx of Ca^2+^ has been associated with the transduction, processing, and modulation of pain signals. This suggests that the analgesic effect of BMSCs may be due to the inhibition of the IL-33/ST2 signaling pathway, which involves the influx of Ca^2+^ and is regulated by miR-547-5p. However, the downstream signaling cascade following IL-33/ST2 inhibition in neurons that ultimately leads to reduced neuropathic pain still needs further exploration.

It is also possible that BMSCs may act on the IL-33/ST2 signaling pathway in immune cells such as macrophages or microglia and exert anti-inflammatory action. In response to tissue damage, IL-33 is released, which acts on MyD88 in cells expressing ST2, activating downstream signaling cascades that ultimately lead to the production of pro-inflammatory cytokines via M1 to M2 macrophage polarization. This pathway can also interact with other signaling pathways such as NF-κB and MAPK, which is also reported to be involved in the production of pro-inflammatory cytokines [66]. Therefore, BMSCs may block the IL-33/ST2 pathway, which in turn reduces pro-inflammatory cytokine production and increases anti-inflammatory cytokine production.

Another miRNA that is thought to be involved in the modulation of neuropathic pain is miR-26a-5p. According to Lu et al. [28], placenta-derived MSC (PMSC) extracellular vesicles (EVs) can reduce pain in neuropathic mice by downregulating the Wnt5a/CamkII/NFAT signaling pathway via miR-26a-5p regulation, resulting in anti-inflammatory effects. The study identified miR-26a-5p as the second most highly expressed miRNA in PMSC EVs and demonstrated that it targets the upregulated Wnt5a gene in the spinal cord of neuropathic mice. While the injection of miR-26a-5p agomir into neuropathic mice reduced the expression of Wnt5a, CamkII, NFAT, and pro-inflammatory cytokines, the changes in these markers after treatment with PMSC EVs were not investigated. Therefore, it remains uncertain whether the analgesic effect of PMSC EVs is solely mediated through the Wnt5a/CamkII/NFAT pathway. The study also suggested that the analgesic effect of PMSC EVs may be attributed to a variety of miRNA acting in tandem, activating multiple signaling pathways to reduce neuropathic pain, as the duration of the analgesic effect of miR-26a-5p agomir injection was shorter than that of treatment with PMSC EVs.

#### 4.3.3. Inhibition of Neuron and Astrocyte Activation

In the three studies by Chen et al. [14], Shiue et al. [17], and Yang et al. [34], it was found that astrocyte activation was inhibited, and the production of pro-inflammatory cytokines was downregulated. Shiue et al. [17] also showed the inhibition of neuron activation and the upregulation of IL-10 production.

According to Yang et al. [34], the analgesic effect of UCBMSCs may be due to the inhibition of astrocyte activity via downregulation of the FOS gene, which reduces the release of pro-inflammatory cytokines and increases the release of anti-inflammatory cytokines. The researchers found that downregulating the expression of miR-29a-3p via LncRNA-H19 led to reduced astrocyte activity and a subsequent reduction in the phosphorylation of ERK and glutamate receptors NR1 and NR2B. FOS, the protein encoded by the c-fos gene, is a commonly used marker for neuronal activation and is closely associated with pain processing in the nervous system. However, research has also indicated that the c-fos gene and its protein can be expressed in astrocytes. A decrease in both c-fos and FOS expression in astrocytes suggests a diminished state of activation in these cells. Reactive astrocytes are commonly linked to nerve injury and upregulation in the production of pro-inflammatory cytokines [68]. Therefore, in the in vivo portion of this study, the observed reduction in pro-inflammatory cytokine production following UCBMSC treatment corresponds with the decreased astrocytic activity reflected by the downregulation of c-fos and FOS expression.

Furthermore, it has been recently reported that an inhibitor of ERK kinase inhibited astrocyte activation and regulated the release of pro-inflammatory cytokines in rats with chronic constriction injury [69], which supports the notion that UCBMSCs reduce astrocyte activation and the subsequent release of pro-inflammatory mediators by decreasing the phosphorylation of ERK. Yang et al. [34] also demonstrated that UCBMSC treatment led to a decrease in the phosphorylation of glutamate receptors NR1 and NR2B in the spinal dorsal horn and astrocytes. These receptors, typically located in neurons, play a crucial role in regulating the accumulation of glutamate—a major neurotransmitter—in the synaptic cleft. Reduced phosphorylation of these glutamate receptors, indicating decreased neuronal activity, can result in diminished pain transmission. However, the presence and function of these receptors in astrocytes have only recently begun to be actively investigated, leaving the role of astrocytic glutamate receptors in mediating neuropathic pain and inflammation still unclear. Notably, a study showed that antagonists targeting NR1 and NR2B on astrocytes can decrease LPS-induced calcium release in these cells, subsequently leading to a reduction in the pro-inflammatory cytokine IL-1β [70]. This may help explain the observed decrease in the phosphorylation of NR1 and NR2B in astrocytes as well as the reduced serum levels of pro-inflammatory cytokines following UCBMSC treatment, as indicated by Yang et al. [34].

Additionally, Yang et al. [34] also showed through an in vitro test that FOS was a downstream target of miR-29a-3p, and inhibiting miR-29a-3p inhibited FOS expression. However, the study did not investigate the direct relationship between FOS and the phosphorylation of ERK and glutamate receptors NR1 and NR2B. Therefore, it remains uncertain whether the reduction in phosphorylation of ERK and glutamate receptors NR1 and NR2B is associated with the decrease in FOS expression.

The study by Shiue et al. [17] revealed an increase in GDNF expression in the DRG of neuropathic rats following treatment with UCMSC-derived exosomes. Exosomes can affect target cells through interactions between their receptors and those of the target cells, leading to the activation of intracellular signaling cascades, or by releasing their contents into the target cell after fusion or internalization [71]. Therefore, the upregulation of GDNF observed in this study could be attributed to either the exosome-stimulated release of GDNF by target cells or a direct release of GDNF by the exosomes themselves. GDNF treatment has been shown to inhibit the upregulation of Nav1.3 [72]. Nav1.3 are voltage-gated sodium channels crucial for generating action potentials in neurons, and they are significantly upregulated in sensory neurons following nerve injury. An increase in the expression of the Nav1.3 channels is accompanied by rapid repolarization of the excitatory potential, leading to persistent abnormal discharge and resulting in the manifestation of neuropathic pain. Therefore, the reduction in pain-like behaviours in rats after treatment with UCMSC exosomes shown by Shiue et al. [17] may be attributed to a decrease in ectopic activity and the subsequent neuronal firing, owing to GDNF’s inhibitory effect on Nav1.3 upregulation. In addition to this, GDNF can be released by both neurons and glial cells, and in disease models, neuroinflammation can lead to overexpression of GDNF by glial cells like astrocytes or macrophages/microglia [73]. Since Shiue et al. [17] showed that astrocytes and neurons were inhibited, it is possible that the upregulated GDNF may have originated from other macrophages/microglia. Previous studies have reported that GDNF secreted by activated macrophages/microglia can promote axonal sprouting and locomotor recovery after nerve injury. Thus, the upregulated GDNF observed in this study may serve to promote axonal regeneration following spinal nerve ligation-induced injury in rats. However, before we can determine the role of GDNF in mediating neuropathic pain, it is essential to identify which cells are responsible for its upregulation.

#### 4.3.4. Reduction of Oxidative Stress

Reactive oxygen species (ROS) and reactive nitrogen species (RNS) are molecules that play a crucial role in various physiological processes at normal levels. However, the production and removal of ROS/RNS must be in balance, and this process is tightly controlled via redox signaling. Endogenous antioxidant enzymes such as superoxide dismutase (SOD) and transcription factors like nuclear factor erythroid 2-related factor 2 (Nrf2) help regulate redox homeostasis. Studies using neuropathic pain models have demonstrated that the administration of ROS donors leads to nociception, while reducing ROS levels through ROS scavengers or antioxidant mimetics has the opposite effect [74]. The accumulation of ROS due to oxidative stress has been associated with the enhancement of excitatory signaling, which can lead to both peripheral and central sensitization, leading to neuropathic pain. In the study by dos Santos et al. [23], neuropathic mice showed elevated levels of oxidative stress markers, nitrite, and malondialdehyde (MDA) in the spinal cord. However, treatment with BMSCs resulted in the downregulation of both markers along with the upregulation of Nrf2 and SOD levels. Therefore, BMSCs may reduce neuropathic pain by upregulating Nrf2 expression, which, in turn, promotes the production of antioxidant and detoxification enzymes, ultimately reducing oxidative stress.

In two separate studies [12,32], MSC treatment was found to downregulate the expression of inducible nitric oxide synthase (iNOS), which is responsible for the production of nitric oxide (NO), a reactive nitrogen species (RNS), in neuropathic pain. Additionally, Wu et al. [32] found that the serotonin receptor 5-HT3A was also downregulated after MSC treatment. It has been reported in several studies that blocking this receptor via its antagonist reduces oxidative stress in the brain [75]. However, the exact mechanism underlying the effect of MSCs on iNOS and 5-HT3A remains unclear and requires further investigation.

#### 4.3.5. Other Pathways

Di Cesare Mannelli et al. [16] proposed that ADMSCs could alleviate neuropathic pain by inhibiting the production of VEGF-A and its isoform VEGF-A165b. The role of VEGF-A and its isoforms in mediating neuropathic pain is still uncertain, as studies have suggested that they may have both pro-nociceptive and anti-nociceptive effects [76]. In the study by Di Cesare Mannelli et al. [16], VEGF-A and its isoform VEGF-A165b were found to have a pro-nociceptive role, contributing to hypersensitivity in rats. Treatment with ADMSCs or antibodies targeting VEGF-A and its isoform resulted in neuropathic pain reduction in rats, which was consistent with a decrease in the levels of VEGF-A and VEGF-A165b.

Another proposed pathway involves the inhibition of synaptophysin, a presynaptic vesicle protein that is associated with synaptic transmission. Studies have reported that overexpression of synaptophysin leads to an increase in the release of neurotransmitters such as glutamate. Furthermore, upregulation of synaptophysin has been observed after sciatic nerve injury [77], which is also reported by Chiang et al. [15]. In their study, treatment with amniotic-fluid MSCs resulted in a downregulation of synaptophysin in neuropathic rats after chronic constriction injury. However, the exact mechanism by which a reduction in synaptophysin levels leads to a reduction in neuropathic pain is still unclear. It is possible that blocking synaptophysin may reduce the release of neurotransmitters, which results in a decrease in pain transmission between the central and peripheral nervous systems. Further research is necessary to fully understand this process.

**Figure 3:**
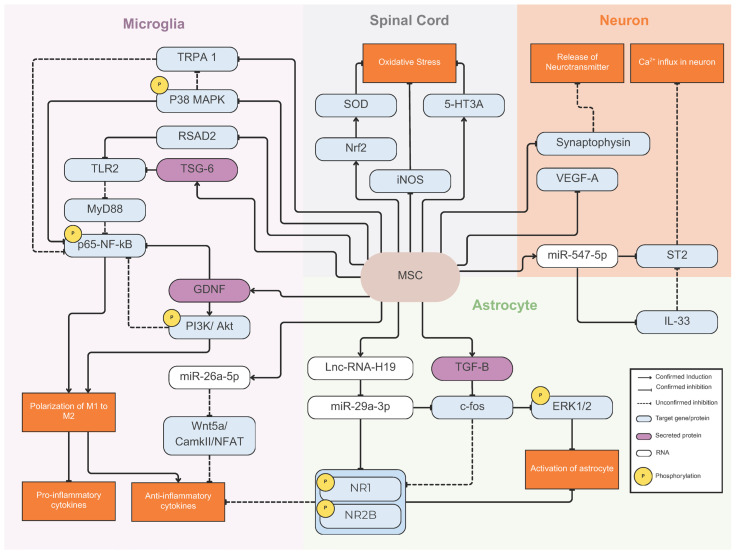
Proposed mechanisms underlying the analgesic and anti-inflammatory effects of mesenchymal stem cells (MSCs) in neuropathic pain.

To summarize, MSCs may help alleviate neuropathic pain and inflammation through various mechanisms. First, they may exert anti-inflammatory effects by promoting microglia to shift from the pro-inflammatory M1 type to the anti-inflammatory M2 type. Second, MSCs may regulate pain-related gene expression in neurons and glial cells via microRNAs found in the extracellular vesicles (EVs) that they release. Additionally, MSCs may inhibit the activation of both neurons and glial cells and reduce oxidative stress by enhancing the production of antioxidant enzymes. These diverse mechanisms highlight how MSCs can modulate neuropathic pain by targeting different cell types and processes involved in pain perception and inflammation. However, as these mechanisms have been studied individually across various animal models and modes of injuries (chemical/mechanical), using difference sources of MSCs and routes of delivery, it remains to be seen whether MSCs act through the same mechanism, either independently or concurrently, collectively contributing to the reduction of neuropathic pain and inflammation. The potential mechanisms of action underlying MSCs’ role in mediating neuropathic pain and inflammation are illustrated in Figure 3.

### 4.4. Do MSCs Secrete Pain and Inflammatory Mediators?

Pain mediators are substances that initiate and regulate specific responses or reactions related to pain. These include ACTH, glucocorticoids, vasopressin, oxytocin, catecholamines, brain opioids, angiotensin II, endorphin/enkephalin, VIP, substance P, eicosanoids (such as prostaglandins and leukotrienes), tissue kininogens (like bradykinin), histamine, serotonin, potassium, and proteolytic enzymes [78]. However, none of the 23 studies reviewed reported direct secretion of pain mediators by MSCs.

Inflammatory mediators include cytokines, histamine, prostaglandins, and leukotrienes. Similarly, none of the 23 studies reviewed reported direct secretion of inflammatory mediators by MSCs. However, most studies did report changes in the expression of pro- and anti-inflammatory cytokines after MSC treatment, which was highly associated with upregulation of the M2 phenotype macrophage/microglia. This suggests that macrophages/microglia are primarily responsible for the changes in cytokine expression. However, the polarization of macrophages/microglia from M1 to M2 must be regulated by some chemical mediator(s), and since this process is upregulated after MSC treatment, it is likely due to the release of chemical mediator(s) by MSCs.

One study [79] showed that inflammation-primed MSCs exhibit higher expression of chemotactic genes, which attract immune cells. Therefore, it is plausible that MSCs secrete inflammatory mediators that regulate the polarization of microglia/macrophages from the M1 to M2 phenotype as well as other processes that are involved in the regulation of inflammation.

### 4.5. MSCs vs. MSC-Secreted Products

Extracellular vesicles (EVs) are lipid-bound vesicles secreted by various cell types, including MSCs, and play an important role in intercellular signaling. EVs are usually categorized into three main subtypes—microvesicles, exosomes, and apoptotic bodies—based on their biogenesis, size, and content. It has become increasingly clear recently that MSCs can alleviate neuropathic pain through paracrine mechanisms, mediated by their secretome, particularly the content of EVs.

Among the four studies that utilized MSC-secreted products, Lu et al. [28] demonstrated the longest analgesic effect of at least 27 days after a single intrathecal injection of PMSC-derived EVs at a dose of 5 µg total protein amount. The shortest analgesic effect reported was up to 5 h after a single intrathecal injection of UCMSC-derived exosomes at a dose of 1.2 µg total protein amount [17].

In the study by Shiue et al. [17], a single injection of exosomes derived from UCMSCs, administered at a dose of 12 µg total protein amount, produced an analgesic effect within 15 min that lasted up to 24 h. Notably, repeated hourly injections of the same dosage for 7 days sustained the analgesic effect for an additional 7 days post-treatment. By comparison, Chen et al. [14], using UCMSCs in the same animal model, achieved a similar 7-day analgesic effect after just a single injection of 1 × 10^6^ cells. Although direct comparison between these studies is limited by differences in dosage quantification—MSCs are typically measured by cell count, whereas exosomes are quantified based on total protein amount—the findings suggest that exosome-based therapies may require continuous or repeated dosing to achieve sustained pain relief. These results also emphasize the need for a standardized approach to exosome quantification, ideally based on the number of parent cells required to produce a specific exosome yield (functional unit). Such a standard would facilitate a more accurate comparison between MSC-based and exosome-based therapies for neuropathic pain.

Additionally, Teng et al. [19] demonstrated that MSC lysates injected intrathecally provided robust and almost instant pain relief in their CCD rat model. However, the pain returned to pre-injection levels after two days. Unlike EVs or MSC lysates, MSCs are living cells capable of sensing and responding to changes in the local microenvironment, especially during tissue injury or inflammation. This enables them to modulate their secretion of signaling molecules in real time according to the requirements of the damaged tissues. In contrast, EVs cannot modify their content after release and lose functionality once their cargo is delivered to the target cells. This passive and limited nature of EVs likely explains the short-term analgesic effect observed in the studies using MSC-secreted products, emphasizing the need for repeated administration of MSC-secreted products such as EVs to maintain an analgesic effect. Despite these limitations, the use of MSC-secreted products does offer some advantages over MSC administration. For example, it can reduce the risk of cell rejection, tumorigenicity, and emboli formation, which have been associated with MSC transplantation. In addition to this, MSC-secreted products are more stable for storage, which is an important factor for commercialization [80].

Additionally, the lack of standardized methods for characterizing EVs based on their content and biological function may contribute to the variability observed across different studies. While current efforts mainly focus on optimizing EV isolation methods to reduce contamination [81], it is equally important to establish standardized protocols for categorizing EVs according to their protein profiles and functional properties. The molecular content of EVs is highly variable and directly influences their biological activity. This variability may result from several factors, including the cellular origin, physiological state of the parent cells, and environmental conditions [82]. For instance, EVs derived from different tissue sources display distinct protein profiles and functional effects: BMSC-derived EVs are associated with the regulation of hematopoiesis and angiogenesis, whereas UCMSC-derived EVs exhibit neuroprotective and anti-inflammatory properties [83]. Furthermore, sudden environmental changes—such as shifting cells to serum-free conditions prior to EV isolation to eliminate serum-derived EVs—can induce cellular stress and change both their metabolism and secretome profile [81]. Notably, studies have reported significant differences in the content of EVs, not only between biological replicates but also among technical replicates [84]. Therefore, developing robust classification systems that group EVs by protein composition and associated biological function is essential for elucidating their therapeutic effects and reducing inconsistencies across EV-related studies.

Similarly, MSC heterogeneity arising from different tissue sources and culture protocols also contributes to the variability in therapeutic outcomes, as demonstrated by the lack of MSC efficacy in studies by Schäfer et al. [13] and Roh et al. [30]. Therefore, establishing clear characteristics and functional markers in MSCs will be required to meet the desirable therapeutic outcome. Moving forward, determining the negative factors associated with adverse effects will be equally important in the release criteria for both MSCs and EVs to ensure quality and safety. Hence, the emphasis on establishing a safe dosage remains a primary focus in clinical trials, with the consideration that repeated dosing may be necessary to achieve optimal therapeutic effects based on individual patient responses.

### 4.6. Clinical Translation

Four clinical trials have been published that assessed the analgesic effect of MSCs in reducing neuropathic pain. The summarized details of these clinical trials are presented in Table 4.

Four clinical trials were conducted to evaluate the therapeutic effects of MSCs on patients with spinal cord injury. Only one of the clinical studies [88] is a randomized controlled trial. The other three studies [85,86,87], conducted by the same research group, are not randomized and lack placebo or control groups. Among the common parameters assessed in all four trials was neuropathic pain. Three of the studies utilized bone marrow-derived MSCs (BMSCs), while the remaining study used Wharton-jelly MSCs (WJMSCs). The administration of the treatment involved delivering MSCs into the spinal cord of patients with spinal cord injury, with dosages ranging from 10 × 10^6^ to 300 × 10^6^ cells per patient. A single injection of either 10 × 10^6^ or 300 × 10^6^ cells resulted in a similar reduction of neuropathic pain by 6 months post-treatment [86,88], suggesting that a dose of 10 × 10^6^ cells may be sufficient to achieve a therapeutic effect. However, neither study reached statistical significance. In a separate study [87], patients received three injections of 100 × 10^6^ cells each (totalling 300 × 10^6^ cells per patient). Notably, almost all patients showed a significant reduction in neuropathic pain after just the first dose, and some even experienced complete resolution of neuropathic pain after the second dose, suggesting that 200 × 10^6^ cells may be sufficient for complete neuropathic pain relief. Collectively, these findings suggest that MSC therapy can provide relief of neuropathic pain, with some patients experiencing improvements as early as two months post-treatment and complete resolution by seven months post-treatment. Despite the varying treatment regimens in each study, initial findings from clinical trials show that MSCs may significantly reduce persistent pain in SCI patients. However, further clinical trials are needed, which should include a larger number of patients and encompass those suffering from neuropathic pain arising from different types of nerve injuries, including both central and peripheral nerve injuries. Such comprehensive trials are essential to provide a better understanding of the potential benefits of MSC treatment for neuropathic pain in various patient populations and to determine whether such treatments can be recommended.

Despite the therapeutic potential of MSCs, there are also safety concerns, particularly regarding the risk of tumorigenicity. Continuous in vitro expansion, often necessary to generate enough cells for therapy, can lead to the accumulation of genetic and epigenetic alterations, resulting in a genomic instability that may promote tumor formation. In addition to this, soluble factors secreted by MSCs may promote the growth of resident tumor cells already present in MSC recipients [89]. A meta-analysis [90] of 62 clinical trials involving MSC therapy found no significant association between MSC administration and tumorigenicity. Most adverse events related to MSC therapy were transient fever, administration site adverse events, insomnia, and constipation. However, it is important to note that the longest follow-up in these studies was only 5 years, and longer-term monitoring is needed to fully evaluate the risk of tumorigenicity. While the overall risk is considered low, it remains essential to implement precautionary measures to minimize potential risks. These include limiting in vitro MSC expansion and screening for genetic alterations before using MSCs in therapy.

## 5. Conclusions

Neuropathic pain is a debilitating condition that affects a large number of people worldwide, and the currently available treatments are often inadequate. Therefore, there is an urgent need to develop more effective treatments that address the underlying mechanisms of neuropathic pain. A total of 23 out of the 25 preclinical studies reviewed reported pain reduction following MSC treatment, with one study noting effects lasting up to day 86. Additionally, MSC studies demonstrated a decrease in pro-inflammatory cytokines, such as IL-1B, as early as 24 h after treatment, alongside an increase in anti-inflammatory cytokines by day 7. Notably, in many of these studies, the decrease in neuropathic pain coincides with a reduction in inflammation. Thus, one of the mechanisms through which MSCs exert their effects is by modulating the immune system, which is often associated with neuropathic pain. The exact signaling pathways underlying the immunomodulatory effect of MSCs in neuropathic pain are not yet fully understood, but the evidence suggests that multiple pathways are involved and that they can interact with each other. Current evidence also suggests that inflammation commonly precedes neuropathic pain, but its resolution does not always eliminate pain, indicating the complexity of the underlying mechanisms of neuropathic pain. Additionally, MSC heterogeneity—including differences in secreted factors—may affect therapeutic outcomes. Future research should focus on defining clear characteristics and functional markers to enhance the reliability and efficacy of MSC-based therapies for the treatment of neuropathic pain.

## Figures and Tables

**Table 1:** Therapeutic potential of MSCs in animal models of neuropathic pain.

Author	Animal, Disease Model	Source of MSC	Route of Administration	Treatment Regimen (Dosage, Frequency)	Findings
**Peripheral nerve injury**					
[10]	Male C57BL/6N mice, spared nerve injury (SNI)	Bone marrow, human	Intrathecal	50,000 cells per injection, once	BMSCs reduced mechanical allodynia starting 6 days post-injection, persisting for 11 days BMSCs reduced thermal hyperalgesia 17 days post-injection (last day of observation) BMSCs reduced activation of astrocyte which in turn reduced production of IL-1β BMSCs reduced over-activation of neural β-galactosidase
[11]	Male CD-1 mice, spared nerve injury (SNI)	Bone marrow, human	Intravenous	2,000,000 cells per injection, once	BMSCs reduced mechanical allodynia and thermal hyperalgesia starting 7 days post-injection, peaking 26 days post-injection, persisting for 79 days BMSCs induced switching of pro-inflammatory M1 macrophages to anti-inflammatory M2 macrophages, which reduced the production of IL-17 and IL-1β, while increasing production of IL-10: ○ IL-17 mainly produced by T-lymphocytes ○ IL-1β mainly produced by astrocytes
[12]	Male C57BL/6J mice, chronic constriction injury (CCI)	Adipose tissue, human	Intravenous	500,000 cells per injection, twice 1,000,000 cells per injection, twice	1 × 10^6^ ASCs reduced mechanical allodynia and thermal hyperalgesia starting 1 day post-injection and lasting until 21 days post-injection 500 × 10^3^ ASCs reduced mechanical allodynia and thermal hyperalgesia only until 7 days post-injection ASCs reduced production of IL-1β in sciatic nerve starting 1 day post-injection ASCs increased IL-10 production in sciatic nerve starting 7 days post-injection ASCs reduced iNOS production in spinal cord
[13]	Female Sprague-Dawley rats, partial sciatic nerve ligation (PSNL)	Bone marrow, rats	Intrathecal	1,000,000 cells per injection, thrice	BMSCs did not improve mechanical allodynia and thermal hyperalgesia in neuropathic rats BMSC-conditioned medium inhibited LPS-induced upregulation of IL-1β and TNF-α in primary microglia culture
[14]	Male Sprague-Dawley rats, spinal nerve ligation (SNL)	Umbilical cord, human	Intrathecal	1,000,000 cells per injection, once	UCMSCs reduced mechanical allodynia starting 4 days post-injection and lasting for 7 days and thermal hyperalgesia starting 2 days post-injection and lasting for 9 days UCMSCs reduced production of IL-1β and IL-17 while increasing IL-10 production in ipsilateral spinal cord UCMSCs reduced activation of astrocytes and microglia in ipsilateral spinal cord
[15]	Sprague-Dawley rats, chronic constriction injury (CCI)	Amniotic fluid, human	Intravenous	500,000 cells per injection, thrice	AFSCs reduced mechanical allodynia and thermal hyperalgesia, which lasted for 14 days from the initial improvement AFSCs reduced production of TNF-α and macrophage activation while increasing S-100 and neurofilament expression in injured sciatic nerve AFSCs reduced production of synaptophysin and TNF-α in dorsal root ganglion AFSCs reduced TNF-α production and microglia activation in dorsal spinal cord Co-culture of AFSCs with dorsal root ganglion cells reduced production of TNF-α, IL-1β, and synaptophysin
[16]	Male Sprague-Dawley rats, oxaliplatin-induced injury	Adipose tissue, rats	Intravenous	2,000,000 cells per injection, four times	ASCs reduced mechanical allodynia and thermal hyperalgesia for 1–5 days per injection ASCs reduced VEGF production
[17]	Male Sprague-Dawley rats, spinal nerve ligation (SNL)	Exosome, umbilical cord, human	Intrathecal	1.2 μg or 6 μg or 12 μg of total protein, once Using an osmotic pump: 1.2 μg total protein/hour, continuous for 7 days	Single injection of Exo-UCMSC, 3 days post-surgery ○ Significantly reduced mechanical allodynia and thermal hyperalgesia after 15 min of injection for all groups and lasted until 5 h post-injection for 1.2 μg group and 24 h for both 6 μg and 12 μg groups ○ The 12 μg group showed a nearly complete absence of mechanical allodynia and thermal hyperalgesia 2–4 h post-injection Single injection of Exo-UCMSC, 8 days post-surgery ○ All dosage groups significantly reduced mechanical allodynia: ▪ 1.2 μg: 30 min to 5 h ▪ 6 μg: 15 min to 6 h ▪ 12 μg: 15 min to 24 h ○ All dosage groups significantly reduced thermal hyperalgesia: ▪ 1.2 μg: 30 min to 5 h ▪ 6 μg: 15 min to 9 h ▪ 12 μg: 15 min to 24 h Continuous injection via osmotic pump, 0–7 days post-surgery ○ Significantly reduced mechanical allodynia and thermal hyperalgesia, from 1 day post-surgery until 14 days post-surgery Continuous injection via osmotic pump, 4–11 days post-surgery ○ Significantly reduced mechanical allodynia and thermal hyperalgesia, from 5 days post-surgery until 18 days post-surgery Exo-UChMSCs inhibited glial activation in both spinal cord and dorsal root ganglion Exo-UCMSCs inhibited neuron activation in both spinal cord and dorsal root ganglion Exo-UCMSCs increased production of IL-10 and GDNF in dorsal root ganglion
[18]	Male C57BL/6 mice, chronic constriction injury (CCI)	Bone marrow, unknown	Intrathecal	250,000 cells per injection, once	BMSCs reduced mechanical allodynia starting 1 day after injection, for 14 days BMSCs reduced thermal hyperalgesia starting 3 days after injection, for 2 days BMSCs inhibited upregulation of CCI-induced phosphorylation of ERK1/2 in the dorsal root ganglion (during early and late treatment) and in the spinal cord (during late treatment) BMSCs upregulated TGF-β1 production in dorsal root ganglion during early treatment Analgesic effect of BMSCs is due to TGF-β1: ○ Addition of TGF-β1 Ab (TGF-β1 neutralizing antibody) inhibited analgesic effect of BMSC
[19]	Male Wistar rats, chronic compression of dorsal root ganglion (CCD)	Bone marrow, Wistar rats	Intrathecal	1,000,000 cells per injection, twice	BMSCs reduced paw withdrawal starting 1 day after injection BMSCs reduced expression of P2X4 in spinal cord of rats BMSCs lysate reduced expression of P2X4 in microglia culture TNP-ATP (P2X4 antagonist) reduced paw withdrawal in untreated group
[20]	Male Sprague-Dawley rats, chronic constriction injury (CCI)	Bone marrow, rats	Intravenous	1,000,000 cells per injection, unknown	BMSCs reduced mechanical allodynia and thermal hyperalgesia BMSCs increased miR-547-5p expression, which reduced the production of IL-33 and ST2 IL-33 was co-localized with astrocytes, whereas ST2 was co-localized with neurons
[21]	Sprague-Dawley rats, deafferentation (transection of spinal dorsal root)	Bone marrow, rats	Dorsal root entry zone	Unknown, once	BMSCs reduced self-mutilation and autotomy BMSCs secreted GDNF, which decreased NF-κB activation while increasing PI3K/AKT pathway activation in microglia, resulting in a change in microglia phenotype from M1 to M2 and subsequent reductions in IL-1β and TNF-α while increasing IL-10 production
[22]	Male Sprague-Dawley rats, chronic constriction injury (CCI)	Bone marrow, rats	Intrathecal	5,000,000 cells per injection, once	BMSCs reduced mechanical allodynia and thermal hyperalgesia starting 2 days after injection, persisting for 11 days. TSG-6 is secreted by BMSCs and inhibits the activation of the TLR2/MyD88 pathway in microglia, which reduces the nuclear translocation of NF-κB and the production of IL-1β, IL-6, and TNF-α
[23]	Male C57BL/6N mice, oxaliplatin-induced sensory neuropathy (OISN)	Bone marrow, C57BL/6N mice	Intravenous	1,000,000 cells per injection, once	BMSCs reduced mechanical allodynia and thermal hyperalgesia for 28 days BMSCs increased production of TGF-β and IL-10 in spinal cord BMSCs increased gene expression of Nrf-2 and SOD while reducing spinal level of MDA and nitrate
[24]	Male C57BL/6N mice, resiniferatoxin-induced post-herpetic neuralgia (PHN)	Bone marrow, unknown	Intravenous	1,000,000 cells per injection, once	BMSCs reduced mechanical allodynia starting 10 days after injection, persisting for 25 days BMSCs reduced p-P38 and p-P65 level in spinal cord BMSCs reduced expression of TRPA1 in spinal cord and dorsal root ganglion TRPA1 antagonist reduced level of p-P65 in spinal cord BMSCs reduced microglial activation and PKCγ expression in spinal cord PKCγ were co-localized with microglia BMSCs reduced CGRP expression in spinal cord BMSCs reduced expression of p-P38, p-P65, and TRPA1 in LPS-stimulated microglia
[25]	Male Wistar rats, chronic constriction injury (CCI)	Conditioned-medium, bone marrow, rats	Intraperitoneal	1 mL of conditioned medium per injection, thrice	Mechanical allodynia and thermal hyperalgesia reduced starting 4 days after injection, persisting for 12 days Conditioned medium of BMSCs reduced expression of P2X4 and P2X7 in spinal cord
[26]	Male and female Balb/C mice, chronic constriction injury (CCI)	Tonsil, human	Intramuscular	1,000,000 cells per injection, once	TMSCs reduced mechanical allodynia starting 3 days after injection, persisting for 6 days TMSCs reduced macrophage infiltration at site of injection TMSCs reduced serum level of TNF-α and IFN-γ TMSCs reduced expression of genes involved in chronic inflammatory response (CCL11, LTA, IL-1β), interleukin-cytokine response (IL-22, IFN-γ), cytokine receptor (CXCR1, CCR7), and humoral immune response (CCL22, NF-κB1)
[27]	Male Sprague-Dawley rats, partial sciatic nerve ligation (PSNL)	Adipose and umbilical cord, human	Intravenous	5,000,000 cells per injection, once	ASCs reduced mechanical allodynia starting 1 day post-injection, lasting for 8 days UCMSCs reduced mechanical allodynia starting 3 days post-injection, lasting for 6 days ASCs and UCMSCs both improved weight distribution of hind paws for the same duration of time ASCs and UCMSCs reduced neuronal damage UCMSCs reduced demyelination of injured nerve ASCs and UCMSCs reduced macrophage accumulation in dorsal root ganglion
[28]	Male C57BL/6N mice, spared nerve injury (SNI)	Small extracellular vesicles, placenta, human	Intrathecal	5 μg of total protein per injection, once	EVs of PMSCs reduced mechanical allodynia starting 1 day after injection, persisting for 27 days EVs of PMSCs reduced microglial activation in spinal dorsal horn miR-26a-5p is the second highest expressed miRNA in PMSC-derived EVs Single injection of miR-26a-5p agomir to SNI mice ○ Reduced thermal hyperalgesia for 21 days ○ Reduced microglial activation ○ Reduced production of IL-1β, IL-6 and TNF-α ○ Reduced gene expression of Wnt5a in spinal cord ○ Reduced expression of CaMKII and NFAT in spinal cord Injection of Foxy5 (Wnt5a mimic peptide) reversed the effects of miR-26a-5p in SNI mice
[29]	Male Sprague-Dawley rats, chronic constriction injury (CCI)	Exosome, umbilical cord, human	Intrathecal	5 μg of total protein per injection, thrice	Exo-UCMSC treatment: ○ Reduced mechanical allodynia for 4 days ○ Reduced microglial activation in spinal dorsal horn ○ Reduced expression of IL-1β, IL-6, TNF-α, and COX-2 in spinal cord ○ Reduced expression of TLR2, MyD88, and p-P65 in spinal cord ○ Reduced expression of RSAD2 in spinal cord ○ Reduced percentage of TLR2/Iba-1 positive cells In vitro culture of Exo-UCMSCs with LPS-induced BV-2 microglia showed ○ Exo-UCMSCs completely internalized by BV-2 microglia ○ Lesser microglial activation ○ Lower expression of TLR2, MyD88, and p-P65 in BV-2 microglia ○ Lower gene expression of IL-1β, IL-6, TNF-α, and iNOS in BV-2 microglia ○ Lower gene and protein expression of RSAD2 in BV-2 microglia knockdown of RSAD2 gene in BV-2 microglia ○ Reduced microglial activation ○ Reduced expression of TLR2, MyD88, and p-P65
**Central nerve injury**					
[30]	Male Sprague-Dawley rats, spinal cord injury (SCI) hemisection	Umbilical cord blood, human	Spinal cord (direct)	1,000,000 cells per injection, once	UCBMSCs did not improve mechanical allodynia and thermal hyperalgesia in neuropathic rats
[31]	Male C57BL/6N mice, spinal cord injury (SCI) contusion	Bone marrow, C57BL/6N mice	Spinal cord (direct)	200,000 cells per injection, once	BMSCs reduced mechanical allodynia and thermal hyperalgesia starting 11 days after injection, persisting for 28 days BMSCs reduced expression of PKC-γ and phosphorylation of CREB in neurons in spinal dorsal horn BMSCs suppressed activation of p38 MAPK and ERK1/2 in macrophages and microglia in spinal dorsal horn
[32]	Male Institute of Cancer Research (ICR) mice, spinal cord injury (SCI) contusion	Umbilical cord, human	Spinal cord (direct)	300,000 cells per injection, once	UCMSCs reduced mechanical allodynia up to 63 days after injection UCMSCs reduced microglial activation UCMSCs downregulated expression of 5-HT3A and iNOS in spinal cord tissue UCMSCs reduced expression of IL-6 and TNF-α while increasing expression of GDNF in spinal cord tissue
[33]	Female Sprague-Dawley rats, spinal cord injury (SCI)	Bone marrow, rats	spinal cord (direct)	70,000 cells per injection, once	BMSCs reduced mechanical allodynia starting 35 days after injection, persisting for 7 days BMSCs reduced microglial activation BMSCs increased expression of MOR and HTT
[34]	Wistar rats, monosodium iodoacetate (MIA)- induced advanced osteoarthritis (OA)	Umbilical cord blood, unknown	Intravenous, intracavity, intrathecal	Unknown, unknown	UCBMSCs reduced mechanical allodynia on the last day of observation, 21 days post-surgery LncRNA H19 is highly expressed in exosomes of UCBMSC miR-29a-3p was the downstream target gene of LncRNA H19, whereas FOS was the downstream target gene of microRNA-29a-3p UCBMSCs lowered serum level of IL-1, IL-2, IL-6, MCP-1, and TNF-α while increasing IL-10 serum level UCBMSCs reduced expression of miR-29a-3p and FOS mRNA in spinal dorsal horn UCBMSCs reduced phosphorylation of NR1, NR2B, ERK and PKCγ in spinal dorsal horn LncRNA H19 and miR-29a-3p inhibitor reduced level of FOS and phosphorylation of NR1, NR2B, ERK, and PKCγ

**Table 2:** Details on MSC administration.

Author	Source of MSC	Route of Administration	Dosage		Concentration (Cells/µL)	Frequency
			Cells/Injection	Cells/Kg Body Weight		
[10]	Bone marrow, human	Intrathecal	50,000	1.25 × 10^6^–1.43 × 10^6^	10,000	Once
[11]	Bone marrow, human	Intravenous	2,000,000	1.25 × 10^6^–1.43 × 10^6^	20,000	Once
[12]	Adipose tissue, human	Intravenous	500,000	20 × 10^6^	2500	Twice
			1,000,000	40 × 10^6^	5000	Twice
[30]	Umbilical cord blood, human	Spinal cord (direct)	1,000,000	5 × 10^6^–5.6 ×10^6^	100,000	Once
[13]	Bone marrow, rat	Intrathecal	1,000,000	-	66,000	Thrice
[31]	Bone marrow, C57BL/6 mouse	Spinal cord (direct)	200,000	7.3 × 10^6^	66,667	Once
[14]	Umbilical cord, human	Intrathecal	1,000,000	4.5 × 10^6^	50,000	Once
[15]	Amniotic fluid, human	Intravenous	500,000	1.67 × 10^6^–2 × 10^6^	2500	Thrice
[16]	Adipose tissue, Wistar rat	Intravenous	2,000,000	8 × 10^6^–10 ×10^6^	5000	Four
[18]	Bone marrow, unknown	Intrathecal	250,000	12.5 × 10^6^	-	Once
[19]	Bone marrow, Wistar rat	Intrathecal	1,000,000	5 × 10^6^–5.56 × 10^6^	66,667	Twice
[20]	Bone marrow, rat	Intravenous	1,000,000	5 × 10^6^–5.56 × 10^6^	3330	-
[21]	Bone marrow, rat	Dorsal root entry zone	-	-	-	Once
[32]	Umbilical cord, human	Spinal cord (direct)	300,000	6.67 × 10^6^–8.1 × 10^6^	100,000	Once
[22]	Bone marrow, rat	Intrathecal	5,000,000	20 × 10^6^–25 × 10^6^	500,000	Once
[33]	Bone marrow, rat	Intramedullary	70,000	0.2 × 10^6^–0.28 × 10^6^	-	Once
[23]	Bone marrow, C57BL/6 mouse	Intravenous	1,000,000	40 × 10^6^–50 × 10^6^	10,000	Once
[24]	Bone marrow, unknown	Intravenous	1,000,000	40 × 10^6^–50 × 10^6^	5000	Once
[34]	Umbilical cord blood, unknown	Intravenous, intracavity, intrathecal	-	-	-	-
[26]	Tonsil, human	Intramuscular	1,000,000	40 × 10^6^–50 × 10^6^	10,000	Once
[27]	Adipose and umbilical cord, human	Intravenous	5,000,000	-	5000	Once

**Table 3:** Details on MSC-secreted product administration.

Author	Cell-Free Therapeutics	Species, Gender	Source of MSC	Route of Administration	Dosage		Total Protein Concentration (μg/μL)	Frequency
					Ug Total Protein/Injection	μg Total Protein/Kg of Body Weight		
[17]	Exosome	Sprague-Dawley rats, male	Umbilical cord, human	Intrathecal	1.2	4.8–6	0.12	Once
					6	24–30	0.6	Once
					12	48–60	1.2	Once
[25]	Conditioned medium	Wistar rats, male	Bone marrow, rats	Intraperitoneal	Information unavailable	Information unavailable	Information unavailable	Thrice
[28]	Small extracellular vesicles (sEVs)	C57BL/6N mice, male	Placenta, human	Intrathecal	5	208.3–250	0.5	Once
[29]	Exosome	Sprague-Dawley rats, male	Umbilical cord, human	Intrathecal	5	Information unavailable	0.2	Thrice

**Table 4:** Details on clinical studies on the therapeutic potential of MSCs for reducing neuropathic pain.

Source	Study Design	Treatment	Findings
[85]	phase 2 clinical trial incomplete SCI 10 patients	using bone marrow-derived mesenchymal stem cells (BMSCs) subarachnoid administration 30 × 10^6^ cells, four times (every 3 months)	Based on visual analog score (VAS), four patients experienced neuropathic pain: three patients showed a reduction in neuropathic pain post-treatment, but it was not significant ○ two patients showed disappearance of neuropathic pain: one at 2 months and another at 7 months post-treatment ○ one patient showed a slight decrease at 2 months post-treatment one patient showed no improvement post-treatment
[86]	phase 2 clinical trial syringomyelia due to traumatic SCI 6 patients	using bone marrow-derived mesenchymal stem cells (BMSCs) into the syrinx (cerebrospinal fluid-filled cyst) 300 × 10^6^ cells, once	Based on visual analog score (VAS), three patients experienced neuropathic pain: all patients showed a decrease in neuropathic pain at 6 months post-treatment, but it was not significant
[87]	phase 2 clinical trial chronic SCI 11 patients	using bone marrow-derived mesenchymal stem cells (BMSCs) intrathecal administration 100 × 10^6^ cells, thrice (every 3 months)	Based on visual analog score (VAS), eight patients experienced neuropathic pain: seven patients showed a significant reduction in neuropathic pain post-treatment ○ all showed reduction of neuropathic pain by 4 months post-treatment ○ four patients showed disappearance of neuropathic pain: two patients by 7 months and another two patients by 10 months post-treatment one patient showed no improvement in neuropathic pain post-treatment
[88]	randomized controlled study phase 1/2a clinical trial chronic complete SCI 10 patients	using Wharton jelly mesenchymal stem cells (WJMSCs) intrathecal administration 10 × 10^6^ cells, once	Based on 0–10 numerical rating scale (0 = no pain, 10 = most intense pain imaginable) most patients (number of patients not available) showed a decrease in neuropathic pain at 6 months post-treatment, but it was not significant

## Data Availability

No new data were created or analyzed in this study. Data sharing is not applicable to this article.
